# IL-17 Genetic and Immunophenotypic Evaluation in Chronic Graft-versus-Host Disease

**DOI:** 10.1155/2014/571231

**Published:** 2014-07-21

**Authors:** Renata Gonçalves Resende, Jeane de Fátima Correia-Silva, Tarcília Aparecida Silva, Ulisses Eliezer Salomão, Luciano Marques-Silva, Érica Leandro Marciano Vieira, Walderez Ornelas Dutra, Ricardo Santiago Gomez

**Affiliations:** ^1^Serviço de Estomatologia, Hospital Municipal Odilon Behrens, Rua Formiga 50, 31110-430 Belo Horizonte, MG, Brazil; ^2^Centro Universitário Newton Paiva, Faculdade de Odontologia, Avenida Presidente Carlos Luz 220, 31230-010 Belo Horizonte, MG, Brazil; ^3^Departamento de Clínica, Patologia e Cirurgia Odontológicas, Faculdade de Odontologia, Universidade Federal de Minas Gerais, Avenida Antônio Carlos 6627, 31270-901 Belo Horizonte, MG, Brazil; ^4^Departamento de Odontologia Restauradora, Faculdade de Odontologia, Universidade Federal de Minas Gerais, Avenida Antônio Carlos 6627, 31270-901 Belo Horizonte, MG, Brazil; ^5^Ciodonto-Universidade FACSETE, Rua Itália Pontelo 50, 35700-170 Sete Lagoas, MG, Brazil; ^6^Laboratório Interdisciplinar de Pesquisa Médica, Faculdade de Medicina, Universidade Federal de Minas Gerais, Avenida Alfredo Balena 190, 30130-10 Belo Horizonte, MG, Brazil; ^7^Departamento de Morfologia, Instituto de Ciências Biológicas, Universidade Federal de Minas Gerais, Avenida Antônio Carlos 6627, 31270-901 Belo Horizonte, MG, Brazil

## Abstract

Although interleukin-17 (IL-17) is a recently discovered cytokine associated with several autoimmune diseases, its role in the pathogenesis of chronic graft-versus-host disease (cGVHD) was not established yet. The objective of this study was to investigate the association of *IL17A* and *IL17F* genes polymorphisms and IL-17A and IL-17F levels with cGVHD. IL-17A expression was also investigated in CD4^+^ T cells of patients with systemic cGVHD. For Part I of the study, fifty-eight allo-HSCT recipients and donors were prospectively studied. Blood samples were obtained to determine *IL17A* and *IL17F* genes polymorphisms. Cytokines levels in blood and saliva were assessed by ELISA at days +35 and +100 after HSCT. In Part II, for the immunophenotypic evaluation, eight patients with systemic cGVHD were selected and the expression of IL-17A was evaluated. We found association between recipient *AA* genotype with systemic cGVHD. No association was observed between IL-17A levels and cGVHD. Lower IL-17A levels in the blood were associated with *AA* genotype. In flow cytometry analysis, decreased expression of IL-17A was observed in patients with cGVHD after stimulation. In conclusion, IL-17A may have an important role in the development of systemic cGVHD.

## 1. Introduction

Hematopoietic stem cell transplant (HSCT) represents the definitive immunotherapy for malignancy and immunologic diseases [1, 2]. However, graft-versus-host disease (GVHD) is an important complication of HSCT that limits its success and can be fatal in approximately 15% of the HSCT patients [3]. Acute GVHD (aGVHD) and chronic GVHD (cGVHD) involve distinct pathological process, where the first one has strong inflammatory components and the second one displays more autoimmune and fibrotic features [4].

T lymphocytes play a critical role in the immune response including allograft rejection, graft failure, and GVHD after transplant; understanding the posttransplant immune regulation might help to generate new therapeutic strategies that might overcome these posttransplant problems [5].

Interleukin-17 (IL-17) is a recently discovered cytokine that can be produced by many cells, although its source from CD4^+^ T cells is the most investigated [6]. The Th17 cells are a new lineage of CD4^+^ T helper cells [7] and generate proinflammatory cytokines such as IL-17A, IL-17F, IL-21, IL-22, tumor necrosis factor (TNF), granulocyte macrophage-colony stimulating (GM-CSF), and some chemokines [6]. IL-17A, the original member of this family, was first identified in 1995 [8] and acts predominantly as a chemoattractant. Recent dates have shown that Th17 pathway is associated with cGVHD [6]. Another Th17 cytokine is the IL-17F; it shows the highest overall amino acid sequence identity with IL-17A in the IL-17 family. The genes encoding IL-17A and IL-17F are both located on 6p12 [9]. IL-17A and IL-17F share biological functions and have many proinflammatory effects in a wide variety of cells, including macrophage, endothelial cells, and fibroblasts [10].

In few recent studies, IL-17A has been associated with the pathway of the GVHD and some studies have assessed the association between* IL17A* gene polymorphism and inflammatory diseases, including the cGVHD [6, 11, 12]. However, the role of IL-17F in the pathogenesis of cGVHD was not previously addressed.

So, the aim of the present study was to investigate the association of the* IL17A *and* IL-17F *gene polymorphisms and IL-17A and IL-17F levels with cGHVD as well as to determine the relationship between IL-17A expression by CD4^+^ T cells and the development of the disease.

## 2. Methods

To achieve the proposed objectives, the work was divided into two parts, which include two groups of individuals. In Part 1 we performed the genotypic and phenotypic analyses of the cytokines, IL-17A and IL-17F, and in Part 2 IL-17A immune marker was assessed. cGVHD was determinate according to NIH Consensus Development Project on Criteria for Clinical Trials [13].

### 2.1. Part 1: Genotypic e Phenotypic Evaluation

#### 2.1.1. Subjects

Fifty-eight consecutive allo-HSCT recipients and related donors from Hospital das Clínicas at Universidade Federal de Minas Gerais (HC-UFMG) were deemed eligible and included in this prospective study. The patients were followed between the days −7 and +100 after it, in a total of sixteen weeks.

All the patients were followed from pre-HSCT until the development of cGVHD, so the number of samples included in each essay varied because of patient's death and loss of samples related to the process of collection. Recipients were conditioned for allo-HSCT according to the specific protocols from the Stem Cell Transplant Unit at HC-UFMG and varied according to the type of the disease, disease status, and the previous treatment at the time of transplantation. Cyclosporine, in combination with methotrexate or mycophenolate mofetil, was used for GVHD prophylaxis, whereas 2 mg/kg of methylprednisolone, in combination with cyclosporine, was used for GVHD treatment.

This study protocol was approved by the local Institutional Ethics Committee (ETIC 0124.0.203.000-11). Written informed consent was obtained from all patients.

#### 2.1.2. Subjects and Sample Collection

Blood samples were collected from recipients and donors one week before the HSTC and were submitted for DNA analysis. Saliva samples were collected using cotton swabs on the floor of the mouth, tongue, and labial and buccal normal oral mucosa of the HSCT subjects. Sites with localized injuries were not included. The cotton swabs were removed with a sterile cytobrush, placed immediately in sterile tubes containing 500 *μ*L of Krebs buffer (NaCl 20%, KCl 2%, CaCl_22_H_2_O 2%, MgSO_4_, KH_2_PO_4_, and C_6_H_12_O_6_), and stored at −20°C until processing. Peripheral blood (4 mL) was collected in vacutainer tubes containing EDTA and stored at −70°C until processing.

#### 2.1.3. DNA Isolation

Total genomic DNA was extracted from saliva and blood samples using a QIAamp DNA blood minikit (Quiagen, Valencia, CA, USA) in accordance with the manufacturer's instructions. The final elution of saliva and blood DNA was performed in 50 *μ*L of a specific AE buffer from the kit and stored at −20°C until use.

#### 2.1.4. *IL17A* and* IL17F* Gene Polymorphism Analysis

Recipient and donor* IL17 *197 (A/G) and IL17F 7488 (T/C) polymorphism was assessed by means of polymerase chain reaction (PCR) amplification and digestion. The sequences of PCR primers for* IL17A* were reverse 5′ AACAAGTAAGAATGAAAAGAGGACATGGT 3′ and ant-sense 5′ CCCCCAAATGAGGTCATAGAAGAGAATC 3′ and for* IL17F* were reverse 5′ GTTCCCATCCAGCAAGAGAC 3′ and ant-sense 5′ AGCTGGGAATGCAAACAAAC 3′, as previously described [14, 15]. The PCR was carried out in a total volume of 50 *μ*L, containing approximately 400 ng of DNA, primers (20 pmol/reaction), and 25 *μ*L of premix buffer (Phoneutria Biotecnologia, Belo Horizonte, Brazil). According to the manufacturer, the premix buffer contained 50 mM KCl, 10 mM Tris-HCl pH 8.4, 0.1% triton X-100, 1.5 mM MgCl_2_, deoxynucleoside triphosphates, and 1.25 units of Taq DNA polymerase. PCR products were digested overnight at 37°C with XagI (Fermentas Life Sciences, Vilnius, Lithuania), for IL-17A, and NlaIII (Fermentas Life Sciences, Vilnius, Lithuania), for IL-17F. Visualization of the product was performed in a 6.5% polyacrylamide gel electrophoresis stained with silver.

#### 2.1.5. Detection of IL-17A and IL-17F Levels

To determine the cytokine levels saliva and blood samples were collected in days +35 and +100 after allo-HSCT. The saliva sample was collected in Salivette tubes (Sarstedt AG & Co, Numbrecht, Germany) according to the manufacturer's instructions. The saliva samples were subsequently diluted (1 : 1) in a PBS solution containing protease inhibitors (0.1 mM PMSF, 0.1 Mm benzethonium chloride, 10 mM EDTA, and 0.01 mg/mL aprotinin A) and 0.05% Tween-20 and subsequently stored at −20°C until analysis. The total protein content in the saliva was determined using the Bradford reagent (Sigma, Saint Louis, MO, USA) and the BSA standard (Fermentas Life Sciences, Vilnius, Lithuania). The total protein content was used to correct the IL-17A values for each sample. The serum samples were obtained from venous blood samples and were centrifuged within 2 hours after blood collection and stored at −20°C.

### 2.2. Part 2: Immunophenotyping Evaluation

#### 2.2.1. Subjects

For immunophenotypic evaluation, additional patient selection was performed and 3 different subgroups were established: HSCT patients with systemic cGVHD (*n* = 8), HSCT patients without systemic cGVHD (*n* = 4), and healthy individuals (*n* = 3).

#### 2.2.2. Blood Collection and Cell Stimulation

Nine milliliters (9 mL) of blood was collected from each individual in a vacuum tube containing sodium heparin by a qualified healthcare professional, complying with the rules for the use of sharp instruments in an aseptic environment. Whole blood was subjected to 3 different conditions: media, stimulus with superantigen staphylococcal enterotoxin B (SEB) (120 ng/mL), and stimulus with anti-CD3 plus anti-CD28 (*α*CD3*α*CD28) monoclonal antibody (Abs) (10 *μ*g/mL) for 20 hours. Brefeldin A (at 1 *μ*g/mL) was added during the last 4 hours of culture. After culture, red blood cell lysis was performed using 1x RBC lysis buffer (eBioscience, San Diego, CA, USA) for flow cytometry, as recommended by the manufacturer.

#### 2.2.3. Cell Surface and Intracellular Staining

After lysis, leukocytes from whole blood were stained with fluorescein isothiocyanate- (FITC-) labeled anti-CD4 monoclonal antibody (eBioscience; BD Pharmingen, San Diego, CA, USA) for 20 minutes at 4°C. The cells were then fixed using 2% formaldehyde (Sigma-Aldrich). The fixed cells were permeabilized and stained, using phycoerythrin- (PE-) labeled anti-cytokine monoclonal antibodies for IL-17A (eBioscience, San Diego, CA, USA), IFN-*γ* (eBioscience, San Diego, CA, USA), or Foxp3 (eBioscience, San Diego, CA, USA), FITC- or PE-labeled isotype control antibodies and an unstimulated cell control were included in all experiments. Preparations were acquired on a FACSCanto II (Becton & Dickinson, San Jose, CA, USA). A minimum of 50,000 gated events on the lymphocyte population were acquired for analysis due to the low frequency of positive events being analyzed. The acquisition was processed using the Diva software (Becton & Dickinson).

#### 2.2.4. Flow Cytometry Data Analysis

**Figure 1:**
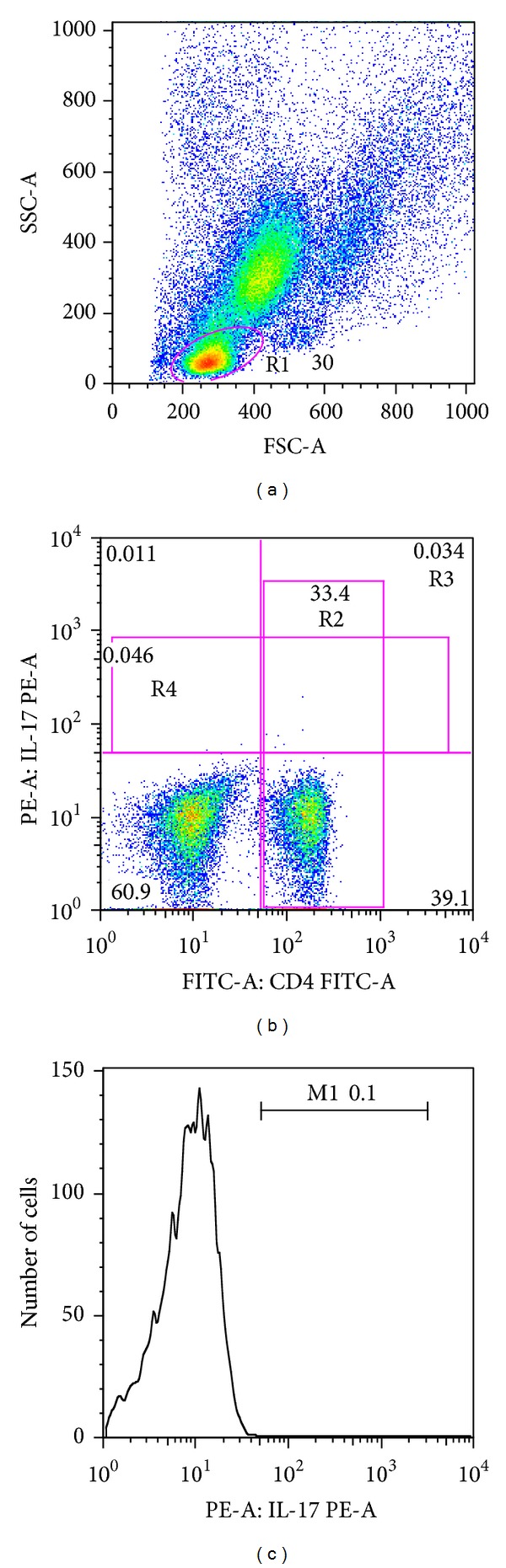
Representative flow cytometry graphs of CD4^+^ lymphocytes expressing intracellular cytokine IL-17A. Flow cytometry dot-plots demonstrate the lymphocyte region (R1) selected (a) and the data analyzed in total CD4^+^ T cells (R2) (b). A single-color histogram (M1) (c) expressing IL-17A in the CD4^+^ T cells was obtained from the R2 region (c). Double-positive CD4^+^IL-17A^+^ in total lymphocytes (R3) and total IL-17A production (R4) were also analyzed in a dot-plot graphic (b).

CD4^+^ T lymphocytes were analyzed for their intracellular expression of IL-17A using the FlowJo program (Tree Star, Ashland, OR, USA). Analyses were performed using a lymphocyte gate (Figure 1(a)). We determined the total expression of IL-17 in the lymphocyte gate (Figure 1(b), R4); we also determined the frequency of Th1 and Th17 cells by analyzing the frequency of CD4^+^IFN-gamma^+^ and CD4^+^IL-17^+^ cells, respectively (Figure 1(b), R3). The expression of IL-17 within the CD4^+^ T cells was assessed by further gating on the total CD4^+^ population (Figure 1(b), R2) and requesting a histogram analysis of such gating (Figure 1(c)). The same strategy was used for IFN-*γ* and Foxp3. Limits for the quadrant markers were always set based on negative populations and isotype controls.

### 2.3. Statistics

The Shapiro-Wilk test was used to determine if samples followed normal distribution. Two independent groups were compared by Student's *t*-test and by the Wilcoxon test. Univariate analyses were performed using the Chi-square, Mann-Whitney, and Friedman tests and Student's *t*-test. The SPSS software was used for the analyses (SPSS Inc., version 19.0, Chicago, IL). Differences were considered significant at *P* < 0.05.

## 3. Results

### 3.1. Part I: Genotypic e Phenotypic Evaluation

#### 3.1.1. Clinical Outcomes

Clinical data from patients and donors is shown in Table 1.

During the monitoring period, the final “*n*” value for the analysis related to IL-17A was 34 and related to IL-17F was 42. The initial “*n*” selected was 58, but over time of patients' followup, losses occurred due to patient death or loss of samples for analysis related to the collection process.

#### 3.1.2. *IL17A* and* IL17F* Gene Polymorphisms in Recipient and Donor of Allo-HSCT and cGVHD

Results of recipient and donor* IL17A *and* IL17F* gene polymorphism and their association with systemic cGVHD are presented in Table 2. We found association between recipient* IL17A AA* genotype and the occurrence of systemic cGVHD (*P* = 0.03).

#### 3.1.3. IL-17A and IL-17F Levels in Blood and Saliva and the Incidence of aGVHD

No association was found between IL-17A or IL-17F levels in the blood or saliva samples and the occurrence of systemic cGVHD (data not shown).

#### 3.1.4. IL-17A Levels in Blood and Saliva and Recipient and Donors* IL17A* Genotypes

Higher IL-17A levels in the blood were observed at day +35 in recipients with* IL17 AG* genotype (*P* = 0.007). No association was found between recipient genotypes and IL-17A levels in saliva or between donor* IL17A* genotypes and IL-17A levels in blood or saliva (Table 3). No association was also found between IL-17F blood or saliva levels and* IL17F *genotypes (data not shown).

### 3.2. Part II: Immunophenotyping Evaluation

#### 3.2.1. Clinical Outcomes

Clinical data from patients and healthy individuals is in Table 4. None of the patients from the group without cGVHD presented the systemic form of the disease.

#### 3.2.2. Expression of IL-17A in Total CD4 and in CD4^+^ T Cells and cGVHD

No statistical difference was found between the expression of total IL-17A and IL-17A^+^CD4^+^ in total T cells, as well as percentage of IL-17A in CD4^+^ T cells and patients with cGVHD (data not shown).

#### 3.2.3. Expression of IL-17A in Total CD4 and in CD4^+^ T Cells and cGVHD after Stimulus

**Figure 2:**
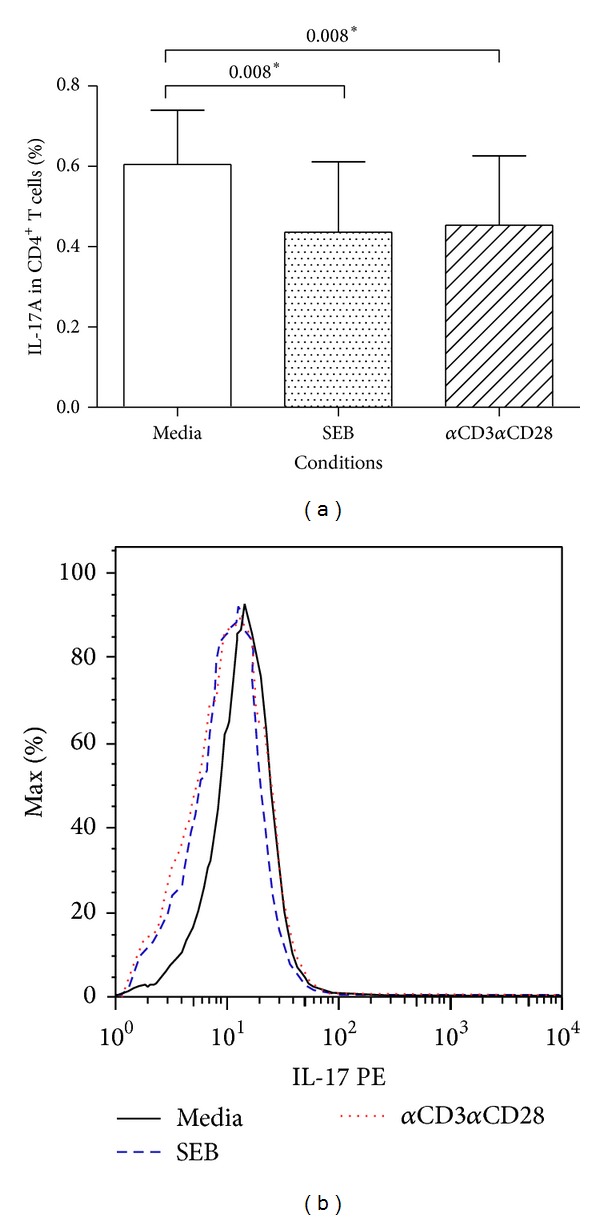
IL-17A expression by CD4^+^ T cells from HSCT patients with cGVHD following culture with SEB and *α*CD3*α*CD28. Whole blood from HSCT patients with cGVHD was maintained in culture without stimulus (media), as well as with SEB and *α*CD3*α*CD28. IL-17A (a) expression in CD4^+^ T cells. The * indicates a *P* value <0.05 between media and stimulus conditions using Wilcoxon's matched pairs test. The overlap histogram graphics represent IL-17 (b) expression in CD4^+^ T cells.

Decreased IL-17A expression was observed in total CD4 T cells of patients with cGVHD after stimulation with SEB (*P* = 0.008) and *α*CD3*α*CD28 (*P* = 0.008) stimulus, compared to media-stimulated control cells (Figure 2(a)). In patients without cGVHD or in healthy individuals, the expression of IL-17A in total CD4 T cells after stimulation with SEB or *α*CD3*α*CD2 was not statistically different than that seen in media-stimulated control cells (data not shown). Overlapping histograms for IL-17A fluorescence are shown in Figure 2(b).

## 4. Discussion

Several studies have been devoted to analyze the role of IL-17, a new discovered cytokine, in the pathogenesis of GVHD [12, 16–18]. However, this is the first work that evaluated both cytokines IL-17A and IL17F, involving the genetic and phenotypic aspects, and investigated the immunophenotypic aspect of IL-17A regarding systemic cGVHD.

In the current study we investigate the involvement of the* IL17A* and* IL17F* gene polymorphism in the cGVHD and our results showed association of* AA* recipient genotypes with the occurrence of systemic cGVHD. Previous studies showed association between G197A polymorphism and several chronic diseases [12], like ulcerative colitis [19] and rheumatoid arthritis [20]. A study reported association between allele* A* of* IL17A* polymorphism and high risk of cGVHD [12]. Until now, no other work was reported in the literature relating* IL17F* polymorphism and GVHD. We did not find association between* IL17F* genotypes and cGVHD; however other authors showed association between* IL17F* polymorphism (T/C 7488) and Behçet disease [21], gastric cancer [14], and bone mineral density [22].

Although our results did not show association between IL-17A or IL-17F levels and the presence of cGVHD, the importance of both in the context of the disease cannot be excluded because we do not understand all the temporal and dynamic factors involved in cGVHD development. The detection of cytokines in a specific time of the disease provides only a snapshot of a more complex process. Th17 pathway has been implicated in the development of acute and cGVHD [6]. Previous preclinical and clinical studies have shown the role of IL-17A in pathogenesis of cGVHD, especially in skin [16, 23].

When we investigated the impact of the* IL17A* polymorphism on the cytokine levels of HSCT patients, we found that recipients with* AA* genotypes showed lower levels of cytokine in the blood in moment +35, compared to the other genotypes. Several polymorphisms in cytokine genes with transcriptional relevance in vitro have been identified [24]. A modification in cytokine production is associated with genetic variations in the promoter region [24–26]. Just one work until now investigated the relationship between* IL17A* polymorphism and IL-17A levels and the authors showed that serum concentration of IL-17A was significantly higher in the presence of allele* A* (*AA* and* AG* genotypes) in a population of Chinese patients with atopic dermatitis [27].

In our study we observed decreased percentage of IL-17A^+^CD4^+^ T cells after stimulus with SEB and *α*CD3*α*CD28. T-cell production of IL-17 induces epithelial, endothelial, and stromal cells to secrete proinflammatory cytokines (IFN-*γ*, IL-6, TNF-*α*, IL-1*β*, and G-CSF) and the levels of these cytokines have been shown to be higher in patients with cGVHD [28]. Therefore, decreased expression of IL-17A in the stimulated cells observed in this work may be caused by the inhibitory effect of others cytokines, like IFN-*γ* [7], which may be relevant to the pathogenesis of cGVHD.

## 5. Conclusion

In conclusion, we demonstrated association between* AA* genotype of recipients with systemic cGVHD and those with lower IL-17A levels and observed that stimulated lymphocytes of patients with cGVHD present decreased expression of IL-17A. Our data indicate that this cytokine could be an important mediator of the cGVHD and may be an important new target for investigation and therapeutics of the disease.

## Figures and Tables

**Table 1:** Clinical characteristics of allo-HSCT patients and donors (*n* = 58).

Parameters	Total (*n* = 58)
Recipient median age in years (range)	31.5 (5–56)
Female gender	25 (43.2%)
Primary disease	
(i) Malignant	
(ii) Chronic myeloid leukemia	9 (15.5%)
(iii) Acute myeloid leukemia	15 (26%)
(iv) Acute lymphoid leukemia	5 (8.6%)
(v) Non-Hodgkin's lymphoma	4 (6.9%)
(vi) Hodgkin's lymphoma	3 (5.1%)
(vii) Other malignancies∗	4 (6.9%)
(viii) Bone marrow failure syndrome∗∗	18 (31%)
HLA match	
(i) HLA matched related	52 (89.6%)
(ii) HLA matched unrelated	4 (6.8%)
(iii) HLA mismatched related	2 (3.6%)
Donor median age in years (range)	35.4 (6–69)
Donor female gender	21 (36.3%)
Conditioning regimen	
(i) BU/CY	20 (34.5%)
(ii) CY +/− ATG or Alemtuzumab	14 (24.2%)
(iii) BU + FLU +/− Alemtuzumab	9 (15.5%)
(iv) CY + FLU +/− Alemtuzumab	8 (13.8%)
(v) MEL + FLU +/− Campath	5 (8.6%)
(vi) Others∗∗∗	2 (3.4%)
Ethnic group	Brazilian mixed population
Source of stem	
(i) Bone marrow	32 (55.2%)
(ii) Peripheral blood stem cells	25 (43.1%)
(iii) Umbilical cord blood	1 (1.7%)

*Myelodysplastic syndrome (*n* = 1), myelofibrosis (*n* = 1), and multiple myeloma (*n* = 2).

∗∗Paroxysmal nocturnal hemoglobinuria (*n* = 2), severe aplastic anemia (*n* = 14), and Fanconi anemia (*n* = 2).

∗∗∗BU/MEL (*n* = 1) Cytarabine/Campath/FLUD (*n* = 1).

BU: busulfan; CY: cyclophosphamide; FLUD: fludarabine; MEL: melphalan; ATG: antithymoglobulins.

**Table 2:** Association between recipient and donor *IL17A *(*n* = 34) and IL-17F (*n* = 42) genotypes and occurrence of systemic cGVHD.

Gene	*IL17A (G197A)* genotype	*n* (%)	Systemic cGVHD		P*	*IL17F* (7488T/C) genotype	*n* (%)	Systemic cGVHD		P*
			A	P				A	P	
Recipient	GG	12 (35.3%)	9	3	**0.03**	CC	10 (23.8%)	7	3	NS
	AG	13 (38.2%)	11	2		CT	18 (42.9%)	9	9	
	AA	9 (26.5%)	3	6		TT	14 (33.3%)	10	4	

Donor	GG	11 (32.4%)	7	4	NS	CC	9 (21.4%)	7	2	NS
	AG	15 (44.1%)	9	6		CT	19 (45.2%)	12	7	
	AA	8 (23.5%)	7	1		TT	14 (33.4%)	7	7	

A: absent; P: present; NS: not significant; ∗Chi-square test.

**Table 3:** Association between saliva IL-17A levels and recipient *IL17A* genotypes (*n* = 34).

	Days	Genotype	*n* valid^&^	IL-17A median	Minimum levels	Maximum levels	P*
Blood levels^a^	+35	GG	14	22.42	0.00	849.80	**0.007**
		AG	13	49.25	0.00	480.14	
		AA	11	0.00	0.00	39.56	
	+100	GG	8	3.59	0.00	178.00	NS
		AG	12	0.00	0.00	116.00	
		AA	8	0.00	0.00	44.02	

Saliva levels^b^	+35	GG	10	0.00	0.00	5.78	NS
		AG	10	1.62	0.00	21.74	
		AA	9	0.00	0.00	11.12	
	+100	GG	9	0.00	0.00	27.00	NS
		AG	8	0.76	0.00	54.12	
		AA	7	0.00	0.00	21.23	

NS: not significant; A: absent; P: present; *Kruskal Wallis test; ^a^pg/mL; ^b^pg/mg protein.

^&^Number of valid samples in each moment.

**Table 4:** Clinical characteristics of recipients with cGVHD (*n* = 8), recipients without cGVHD (*n* = 4), and healthy individuals (*n* = 3) included in the immunophenotypic analysis.

Groups	*n*	Age median	Individuals gender	Donor gender	Source of cells	Media time after HSCT
With cGVHD	8	37.2	Female = 2 (25%)	Female = 1 (12.5%)	BMSC = 2 (25%)	5 years and 8 months
			Male = 6 (75%)	Male = 7 (87.5%)	PBSC = 6 (75%)	
Without cGVHD	4	41.5	Female = 3 (75%)	Female = 2 (50%)	BMSC = 4 (100%)	6 years and 1 month
			Male = 1 (25%)	Male = 2 (50%)	PBSC = 0	
Healthy individuals	3	26.6	Female = 2 (66.7%)	—	—	—
			Male = 1 (33.3%)			

BMSC: bone marrow stem cells; PBSC: peripheral blood stem cells.

## References

[1] Anderson BE, McNiff JM, Jain D, Blazar BR, Shlomchik WD, Shlomchik MJ (2005). Distinct roles for donor- and host-derived antigen-presenting cells and costimulatory molecules in murine chronic graft-versus-host disease: requirements depend on target organ. *Blood*.

[2] van den Brink MR, Burakoff SJ (2002). Cytolytic pathways in haematopoietic stem-cell transplantation. *Nature Reviews Immunology*.

[3] Pasquini MC, Griffith LM, Arnold DL (2010). Hematopoietic stem cell transplantation for multiple sclerosis: collaboration of the CIBMTR and EBMT to facilitate international clinical studies. *Biology of Blood and Marrow Transplantation*.

[4] Blazar BR, Murphy WJ, Abedi M (2012). Advances in graft-versus-host disease biology and therapy. *Nature Reviews Immunology*.

[5] Wood KJ, Bushell A, Hester J (2012). Regulatory immune cells in transplantation. *Nature Reviews Immunology*.

[6] Serody JS, Hill GR (2012). The IL-17 differentiation pathway and its role in transplant outcome. *Biology of Blood and Marrow Transplantation*.

[7] Harrington LE, Hatton RD, Mangan PR (2005). Interleukin 17-producing CD4^+^ effector T cells develop via a lineage distinct from the T helper type 1 and 2 lineages. *Nature Immunology*.

[8] Yao Z, Fanslow WC, Seldin MF (1995). Herpesvirus Saimiri encodes a new cytokine, IL-17, which binds to a novel cytokine receptor. *Immunity*.

[9] Park H, Li Z, Yang XO (2005). A distinct lineage of CD4 T cells regulates tissue in?ammation by producing interleukin 17. *Nature Immunology*.

[10] Shibata K, Yamada H, Nakamura R, Sun X, Itsumi M, Yoshikai Y (2008). Identification of CD25+ *γδ* T cells as fetal thymus-derived naturally occurring IL-17 producers. *The Journal of Immunology*.

[11] Corrêa JD, Madeira MFM, Resende RG (2012). Association between polymorphisms in interleukin-17A and -17F genes and chronic periodontal disease. *Mediators of Inflammation*.

[12] Espinoza JL, Takami A, Onizuka M (2011). A single nucleotide polymorphism of IL-17 gene in the recipient is associated with acute GVHD after HLA-matched unrelated BMT. *Bone Marrow Transplantation*.

[13] Filipovich AH, Weisdorf D, Pavletic S (2005). National Institutes of Health consensus development project on criteria for clinical trials in chronic graft -versus-host disease: I. diagnosis and staging working group report. *Biology of Blood and Marrow Transplantation*.

[14] Wu X, Zeng Z, Chen B (2010). Association between polymorphisms in interleukin-17A and interleukin-17F genes and risks of gastric cancer. *International Journal of Cancer*.

[15] Kawaguchi M, Takahashi D, Hizawa N (2006). IL-17F sequence variant (His161Arg) is associated with protection against asthma and antagonizes wild-type IL-17F activity. *Journal of Allergy and Clinical Immunology*.

[16] Carlson MJ, West ML, Coghill JM, Panoskaltsis-Mortari A, Blazar BR, Serody JS (2009). In vitro differentiated TH17 cells mediate lethal acute graft-versus-host disease with severe cutaneous and pulmonary pathologic manifestations. *Blood*.

[17] Iclozan C, Yu Y, Liu C (2010). T helper17 cells are sufficient but not necessary to induce acute graft-versus-host disease. *Biology of Blood and Marrow Transplantation*.

[18] Yi T, Zhao D, Lin C (2008). Absence of donor Thl7 leads to augmented Thl differentiation and exacerbated acute graft-versus-host disease. *Blood*.

[19] Arisawa T, Tahara T, Shibata T (2008). The influence of polymorphisms of interleukin-17A and interleukin-17F genes on the susceptibility to ulcerative colitis. *Journal of Clinical Immunology*.

[20] Nordang GBN, Viken MK, Hollis-moffatt JE (2009). Association analysis of the interleukin 17A gene in Caucasian rheumatoid arthritis patients from Norway and New Zealand. *Rheumatology*.

[21] Jang WC, Nam YH, Ahn YC (2008). Interleukin-17F gene polymorphisms in Korean patients with Behçet's disease. *Rheumatology International*.

[22] Oishi Y, Watanabe Y, Shinoda S (2012). The IL6 gene polymorphism -634C>G and IL17F gene polymorphism 7488T>C influence bone mineral density in young and elderly Japanese women. *Gene*.

[23] Dander E, Balduzzi A, Zappa G (2009). Interleukin-17-producing t-helper cells as new potential player mediating graft-versus-host disease in patients undergoing allogeneic stem-cell transplantation. *Transplantation*.

[24] Accardo Palumbo A, Forte GI, Pileri D (2012). Analysis of IL-6, IL-10 and IL-17 genetic polymorphisms as risk factors for sepsis development in burned patients. *Burns*.

[25] Crawley E, Kay R, Sillibiurne J (1999). Polymorphic haplotypes of the interleukin-10 5′ flanking region determine variable interleukin-10 transcription and are associated with particular phenotypes of juvenile rheumatoid arthritis. *Arthritis and Rheumatology*.

[26] Schroder O, Laun RA, Roher HD (2003). Heat shock protein 70 genotypes HSPA1B and HSPA1L influence cytokine levels and interfere with outcome after major injury. *Critical Care Medicine*.

[27] Ma L, Xue H-B, Guan X-H (2012). Possible role of Th17 cells and IL-17 in the pathogenesis of atopic dermatitis in Northern China. *Journal of Dermatological Science*.

[28] Chen X, Das R, Komorowski R, van Snick J, Uyttenhove C, Drobyski WR (2010). Interleukin 17 is not required for autoimmune-mediated pathologic damage during chronic graft-versus-host disease. *Biology of Blood and Marrow Transplantation*.

